# Separating decadal global water cycle variability from sea level rise

**DOI:** 10.1038/s41598-017-00875-5

**Published:** 2017-04-20

**Authors:** B. D. Hamlington, J. T. Reager, M.-H. Lo, K. B. Karnauskas, R. R. Leben

**Affiliations:** 1 Center for Coastal Physical Oceanography, Old Dominion University, Norfolk, USA; 2 Jet Propulsion Laboratory, California Institute of Technology, Pasadena, CA USA; 3 Department of Atmospheric Sciences, National Taiwan University, Taipei, Taiwan; 4 Department of Atmospheric and Oceanic Sciences and Cooperative Institute for Research in Environmental Sciences, University of Colorado Boulder, Boulder, Colorado USA; 5 Colorado Center for Astrodynamics Research, University of Colorado, Boulder, USA

## Abstract

Under a warming climate, amplification of the water cycle and changes in precipitation patterns over land are expected to occur, subsequently impacting the terrestrial water balance. On global scales, such changes in terrestrial water storage (TWS) will be reflected in the water contained in the ocean and can manifest as global sea level variations. Naturally occurring climate-driven TWS variability can temporarily obscure the long-term trend in sea level rise, in addition to modulating the impacts of sea level rise through natural periodic undulation in regional and global sea level. The internal variability of the global water cycle, therefore, confounds both the detection and attribution of sea level rise. Here, we use a suite of observations to quantify and map the contribution of TWS variability to sea level variability on decadal timescales. In particular, we find that decadal sea level variability centered in the Pacific Ocean is closely tied to low frequency variability of TWS in key areas across the globe. The unambiguous identification and clean separation of this component of variability is the missing step in uncovering the anthropogenic trend in sea level and understanding the potential for low-frequency modulation of future TWS impacts including flooding and drought.

## Introduction

As the climate continues to warm, there is an expectation for increased variability in the water cycle and associated shifts in precipitation patterns over land, subsequently impacting terrestrial water balance^1–4^. On global scales, such changes in terrestrial water storage (TWS) will be reflected in the water contained in the ocean and will manifest as variability in global sea level^5–7^, establishing a direct link between observations of the global water cycle and sea level. Understanding this link should enable us to separate the long-term trend in sea level from natural variability about this trend. However, achieving this separation has been elusive thus far, and remains a key barrier to improving our understanding of past and present sea level, and subsequently projections of future sea level rise. Specifically, naturally occurring climate-driven TWS variability can temporarily obscure the long-term trend in sea level rise, in addition to modulating the impacts of sea level rise through natural periodic undulation in regional and global sea level^8–11^. Furthermore, with the potential for naturally occurring TWS patterns to change under a warming climate, improving the understanding and representation of this variability in the past and present will aid in future identification of any possible shifts.

With major advances in Earth observing systems over the past decade, it is now possible to study sea level and the global water cycle in tandem on both regional and global scales. Sea level measurements from satellite altimeters have been used along with TWS estimates from the GRACE satellites to establish a relationship between the two sets of observations^11, 12^. As has been demonstrated, reductions of TWS are mirrored by a similar increase of water in the ocean (and vice versa), with the El Nino-Southern Oscillation (ENSO) shown to play a dramatic role in this water exchange on interannual timescales^10, 12, 13^. Analogously, sea surface salinity from *in situ* and space-borne sensors monitor the oceanic component of the global water cycle. On longer timescales, however, the utility of satellite observations is more limited. The GRACE satellites extend back to only 2002, with the associated satellite record of TWS only recently surpassing a decade in length. While the satellite altimeter record began in 1993, questions remain regarding the ability to separate decadal-scale variability from a now 23-year record [e.g. ref. 14]. In a recent study, Reager *et al*.^11^ estimated the climate-driven variability in the GRACE record, finding significant contributions to decadal trends in global TWS and global mean sea level (GMSL), while further identifying how this decadal signal varies across the globe. This result has important implications not only for the study of sea level, showing that such natural climate variability can serve to mask the true underlying rate of GMSL rise (which happened to be higher over the time period from 2002–2014), but also for water exchange between land and ocean, demonstrating that significant trends in TWS can persist over the course of a decade and suggesting extended periods of flooding and drought associated with climate-driven variability. It remains unknown, however, whether this decadal trend can be expected to persist or even reverse in the near future, or alternatively, whether similar trends have been observed in the past. Understanding the timescale upon which this climate-driven signal varies is important for both the interpretation of historical flooding and drought and also in developing plans for climate resilient infrastructure. Similarly, projecting future sea level rise requires a clear representation of the long-term trend, with variability about this trend serving as a source of “noise” that has yet to be fully understood or quantified. These issues cannot be resolved directly by the short satellite records and clearly require the use of longer data records from which decadal variability can be extracted. Our investigation employs a multivariate approach to best understand the decadal variability in the global water cycle and its contribution to historical sea level.

## Data

Here, we create a new sea level reconstruction spanning the time period from 1900 through 2014 that is used to investigate the decadal variability in sea level and TWS. As discussed in Calafat *et al*.^15^, attempting to include the background trend into reconstructions of sea level limits the ability to accurately reconstruct internal climate variability both regionally and globally. To remedy this problem, we reconstruct only the variability about the long-term, background trend. We use a novel bivariate technique that incorporates sea surface temperature (SST) measurements into the reconstruction of sea level to overcome the very limited tide gauge sampling before 1950^16^. This technique involves computing cyclostationary empirical orthogonal functions (CSEOFs) of satellite-measured sea level and SST, performing a simple regression to create spatial patterns in each variable with a common temporal evolution, and subsequently fitting these patterns to tide gauge and historical SST data. Further details are found in Hamlington *et al*.^16^. For the longer TWS record, we use the Global Land Data Assimilation System Version 2 (GLDAS-2) dataset^17^. The TWS data used comes from the sum of the soil moisture and snow water equivalent output variables, and covers the time period from 1948 through 2010. The goal of this study is to leverage a consistent decadal signal in both sea level and TWS that can be attributed to modes of climate variability. To further diagnose the mechanisms for the reconstructed decadal variability in the oceanic and terrestrial accumulation of freshwater, analyses of precipitation (NOAA PREC and PREC/L, 1948–2011) and sea surface salinity (SSS; Hadley EN4.2.0, 1900–2015) are also performed.

## Results

### Linking Regional Decadal Variability in Sea Level and TWS

**Figure 1:**
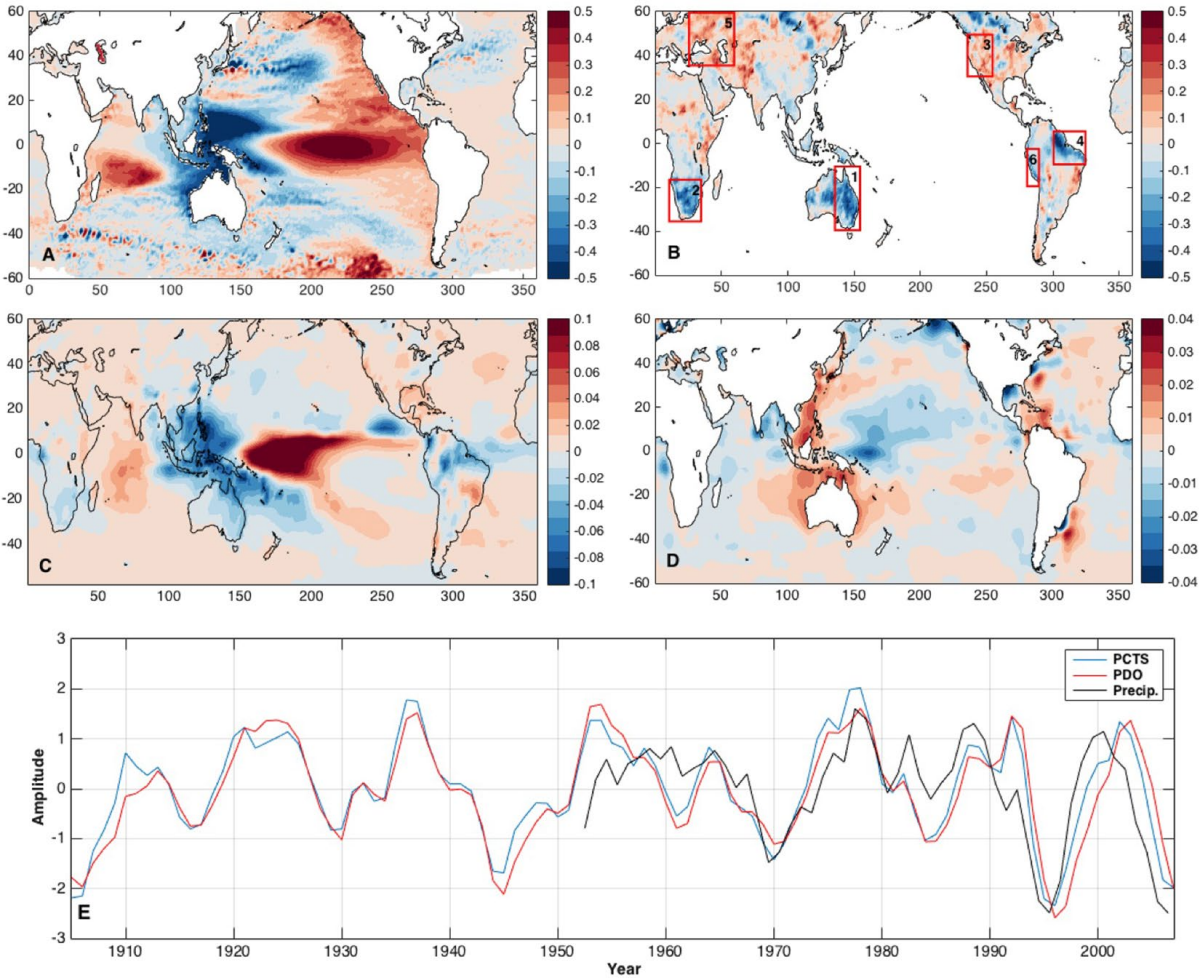
(**A**) First EOF LV of the ten-year trends computed from the sea level reconstruction from 1900 to 2014 (mm/year). (**B**) EOF LV of TWS obtained by projecting the PCTS from the first sea level EOF onto the ten-year trend maps from GLDAS-2 (mm/year). (**C**) EOF LV of precipitation obtained by projecting the PCTS from the first sea level EOF onto the ten-year trend maps from NOAA PREC dataset. (**D**) EOF LV of SSS obtained by projecting the PCTS from the first sea level EOF onto the ten-year trend maps from Hadley EN4.2.0 (psu/year). (**E**) PCTS for the LVs shown in A, B and C, compared to the ten-year trends in the PDO index. The ratio of integrated precipitation over land to integrated precipitation over the ocean is also shown. (Figure was created using MATLAB, R2016b, www.mathworks.com).

We first determined the spatial footprint of the dominant global, decadal trend pattern in sea level (see Methods) by performing an empirical orthogonal function (EOF) decomposition of decadal sea level trends. The resulting spatial pattern (Fig. 1A), termed the loading vector (LV), strongly resembles the pattern associated with the Pacific Decadal Oscillation (PDO)^18^ in the north Pacific, with a similar resemblance to the Interdecadal Pacific Oscillation (IPO) throughout the Pacific. Indeed, the associated time series (Fig. 1E), termed the principal component time series (PC), has a correlation of 0.95 with the time series of decadal trends estimated from the PDO index from 1900 to 2014 computed from the sea level reconstruction, following Cummins *et al*.^19^. Although there is a clear relationship with the PDO index, we do not claim in this study that the decadal variability is being driven by the PDO. Recent studies have re-examined the statistical characteristics of the PDO^20^, describing the PDO not as a single climate mode, but as the result of a combination of processes with different origins (*e*.*g*., tropical and polar) and different time scales (*e*.*g*., interannual and quasi-decadal). As such, any correlation between ocean variability (like sea level, in the case of the present study) and the PDO may be due to common forcing rather than an indication of a direct, dynamical linkage between that variability and PDO itself. Further, the present study deals only with global modes, and while the PDO may provide a good representation for the decadal variability in the Pacific Ocean, it is not suggested here that a statistical mode defined in the north Pacific is physically meaningful for the entire globe. By focusing on global modes obtained with an EOF decomposition, we are not imposing any particular structure or temporal evolution and are looking for a common link or signal that explains decadal variability at locations across the globe. This also means that we are not necessarily looking to explain these global modes physically and instead allow for statistical descriptions of any links obtained through our modal analysis.

To obtain a corresponding mode in TWS, the PC from sea level (Fig. 1E) is projected onto the decadal trend patterns similarly computed from GLDAS-2 from 1948 to 2011. The resulting LV is shown in Fig. 1B, exhibiting similarities to the map of climate-driven trends derived from GRACE in Reager *et al*.^11^, particularly in the negative areas (e.g. Australia, southern Africa, Amazon etc.). The same projection of the PC is performed on decadal trends in precipitation (Fig. 1C) and SSS (Fig. 1D), yielding a number of notable features that independently corroborate the decadal sea level variability while shedding light on its physical mechanisms. In general, the precipitation and SSS patterns correspond closely to the TWS storage pattern shown in Fig. 1B. As a more specific example, during times of decreased TWS in Australia the SSS increases in the surrounding ocean, consistent with the decreased precipitation in the area. There is also a sharp gradient in the decadal SSS pattern in this region, with the opposite sign observed off the east coast of Papua New Guinea. This decadal SSS variability is locally consistent with the precipitation pattern over the oceans (i.e., high decadal SSS anomaly collocated with low decadal precipitation anomaly), which itself is associated with decadal variability in the globally integrated land-ocean precipitation difference. Although the associated pattern of precipitation (Fig. 1C) projects strongly onto the canonical precipitation anomaly pattern associated with El Nino events, the fact that the pattern also emerges at this lower frequency time scale implies an important decadal persistence in the coupled ocean-atmosphere interactions. Furthermore, these coupled interactions are manifest as a proxy for PDO-like variability in *in situ* SSS observations, with a significant correlation (−0.55) between the globally integrated land-ocean precipitation difference and SSS at 167E, 0N from 1948 to 2012. Put simply, salinity just off the coast of Papua New Guinea could potentially be a gauge for the global cycling of freshwater among the oceanic and terrestrial realms, which also drives a large, coherent pattern of decadal sea level variability.

### Contributions to Global Mean Sea Level and Global Water Cycle

**Figure 2:**
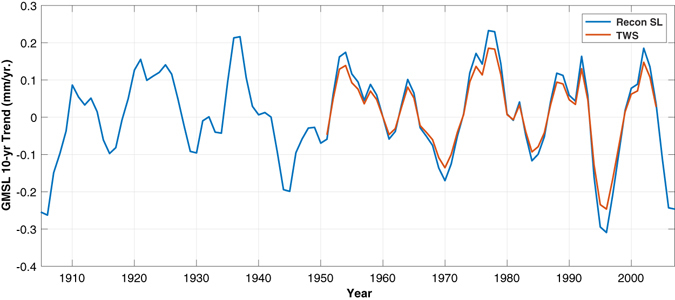
Contribution of the EOF modes shown in Fig. 1 to the 10-year trends in GMSL. The TWS is estimated from the EOF projection onto the GLDAS-2 data (Fig. 1b). Note, the sign of the contribution from the GLDAS-2 EOF has been reversed to reflect the contribution to GMSL and not the contribution to TWS.

In Reager *et al*.^11^, using the GRACE record, the contribution of the total continental water storage (excluding glaciers and ice sheets) to GMSL was found to be −0.33 mm/year over the time period from 2002 to 2014. Using the result shown in Fig. 1, the contribution of the sea level and TWS trend patterns to decadal trends in GMSL can be estimated directly and compared to the Reager *et al*.^11^ result (Fig. 2). Over the time period from 2002 to 2012, the sea level reconstruction-computed EOF contributed −0.25 mm/year to GMSL. Since the GLDAS-2 data extends only to 2010, a similar number cannot be obtained for comparison to GRACE and the sea level reconstruction. We can, however, estimate the GMSL contribution from TWS over the rest of the record. Although the temporal variability of each EOF is required to be the same as a result of the projection of the PC, the GMSL contributions calculated from the sea level reconstruction and GLDAS-2 EOFs are under no such restriction and exhibit excellent agreement throughout the record. The GMSL contributions are roughly 75% of the value computed directly from GRACE, although this is not unexpected given the focus here on only the dominant mode of decadal trend variability. Throughout the time series from 1948 to 2012, the GMSL contribution from the sea level reconstruction and GLDAS-2 agrees remarkably well. Extending the discussion of Fig. 1, changes in precipitation between land and ocean can also be related to this agreement in GMSL and TWS. After integrating the precipitation over land and ocean (separately) on a global scale, a time series is created representing the ratio of land precipitation to ocean precipitation from 1948 to 2012 (Fig. 1E, black; note, this is not computed by projecting the EOF mode onto the precipitation data). The resulting ratio has a significant correlation of 0.74 with the PCTS in Fig. 1E and with the GMSL and TWS in Fig. 2. In other words, secular amplifications of the decadal trends in GMSL are highly correlated with increased precipitation over the ocean and decreased precipitation over land, leading to a net movement of water from land to ocean.

### Decadal Variability in Regional TWS

**Figure 3:**
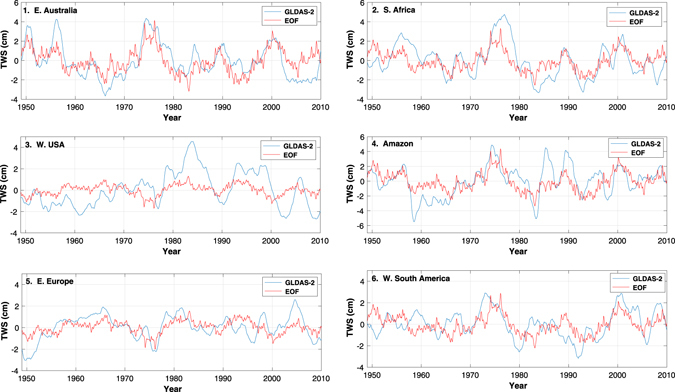
Comparison between GLDAS-2 TWS (after 2-year smoothing) and the contribution of the EOF mode shown in Fig. 1B to TWS. The time series for six separate regions are shown, matching the boxes shown in Fig. 1B.

By projecting the spatial pattern in Fig. 1B back onto the TWS data from GLDAS-2, we can find the time series of TWS variability from 1948 through 2010 associated with the extracted climate signal. Comparing this to the total data from GLDAS-2 allows us to identify the extent to which this lower-frequency mode describes extended periods of enhanced or suppressed moisture. In particular, we select six regions (shown in Fig. 1B) with particularly strong signals in the LV: (1) Australia, (2) southern Africa, (3) western United States, (4) Amazon basin, (5) eastern Europe, and (6) western South America. In Fig. 3, the TWS contribution from the extracted EOF mode was averaged in each box and compared to the total TWS from GLDAS-2 (smoothed with a two-year window). While large, short-term individual events in each location are not captured by the EOF decomposition, extended time periods of high or low moisture agree very well with the total signal. Additionally, these extended time periods agree well with historical accounts and past literature detailing drought and flooding in each location. Examining the time series in more detail, the large signal in the 1970s, for example, clearly illustrates the extended increase in rainfall and subsequent flooding that occurred in Australia^21, 22^, southern Africa^23^ and Amazon Basin^24^ and the reduced rainfall that occurred in the western United States^24^ and eastern Europe^25^. The opposite pattern persisted for much of the early to mid 1990s [e.g. refs 26 and 27]. It is important to note that these time series do not simply track ENSO variability. For example, the exceptionally large signals in the 1970s and early 1990s do not coincide with similarly large ENSO events.

### Impact on the Estimating the Long-term Sea Level Trend

**Figure 4:**
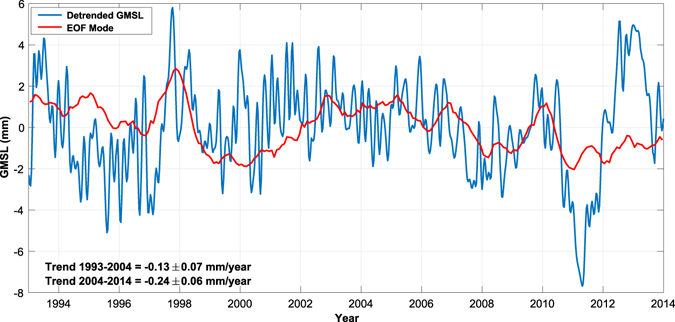
Contribution of the EOF sea level mode to GMSL (red), estimated by projecting spatial pattern (Fig. 1A) on to AVISO satellite altimetry data from 1993–2014. Trends are computed from the EOF mode contribution (red) for the time periods from 1992 to 2004 and 2004–2014. Detrended GMSL computed directly from AVISO (blue) is shown for comparison.

In terms of sea level, extracting periodic variability that may be obscuring the background trend could lead to an earlier identification of an acceleration in sea level that was previously undetectable. To investigate the effect of removing the periodic signal described here, the spatial pattern in Fig. 1A is projected back on to the AVISO sea level observations from 1993 to 2014, and the resulting GMSL time series is compared to the detrended AVISO GMSL (Fig. 4). During the first half of the record (1993 through 2003), the effect of the decadal mode was to lower GMSL by 0.13 +/− 0.07 mm/year, while during the second half (2004 through 2014), the decadal mode lowered the estimated GMSL by 0.24 +/− 0.06 mm/year. In other words, this decadal variability served to both lower the overall trend measured by satellite altimetry and introduce an apparent deceleration in the global mean sea level rise. While this is a relatively small effect in terms of the full trend, with accelerations on the order of hundredths of mm/year^2^ being recently published^28^, the impact of the decadal variability identified here on estimated accelerations is certainly not negligible.

## Discussion and Conclusions

The consistency on global scales between the sea level reconstruction, reanalysis TWS from GLDAS-2, and measures of the oceanic component of the global water cycle demonstrates the ability of multiple datasets to capture physically consistent expressions of decadal climate variability. Furthermore, the relationship between a known climate signal (as represented in sea level in this case) and regional TWS variability has important implications for future water availability in addition to providing better context for the GRACE record. An inherent timescale of persistent, low frequency moisture or dryness has been identified, and related to the occurrence of flooding and drought around the world. In Fig. 1, we further demonstrate the agreement between the decadal climate mode and the PDO index. As discussed above, however, we do not suggest that the PDO is simply driving the TWS trends. The PDO has recently been described as the complex product of several different tropical and extratropical processes rather than a single climate signal [e.g. ref. 20]. Correlations between oceanic and atmospheric variability and the PDO may be due to common forcing rather than an indicator of a dynamical coupling driving an intrinsic PDO. In the present study, this common forcing is likely leading to significant decadal TWS variability and the occurrence of extended flooding and drought periods at many locations across the globe. With an apparent ongoing shift in Pacific decadal variability^29^ this has important implications for TWS in the coming decade and provides context for the GRACE record to date. For example it is likely that as a result of this decadal variability, the next decade will be marked by decreased TWS in Australia, southern Africa and the Amazon basin while the western United States and western Asia will see increased TWS potentially leading to lifting of recent drought conditions. Based on our analysis, these changes on land will be matched by general increase in the global rate of sea level rise.

In general, understanding how Earth’s climate varies on decadal timescales and, especially, the way in which fresh water is passed between different reservoirs within the global water cycle, rightfully remains at the forefront of climate science with wide-ranging implications with regards to understanding future conditions both in the near-term and long-term. As we have seen, internal variability in this system can lead to decadal variability in GMSL that serves to enhance or suppress the underlying long-term trends. It should be noted that we are not suggesting here that all decadal sea level variability is related to TWS, but do find TWS variability to play a significant role in sea level changes on the timescale of a decade. The results demonstrate that precipitation-driven variability in globally integrated quantities can be regional in nature, yet lead to decadal changes in the exchange of water between land and ocean that is measurable on global scales. While the resulting relationship between GMSL, precipitation, SSS and TWS is consistent with first principles of climate dynamics, extracting such a multivariate signal that persists for a decade and undergoes shifts in phase as evidenced by the historical record demonstrates the important linkages in the global water cycle impacting sea level that can now be continuously monitored by satellites.

## Methods

The reconstructed sea level dataset used here is created following the methodology outlined in previous literature^16^, with two notable differences. First, as discussed in Calafat *et al*.^15^, attempting to include the background trend into reconstructions of sea level limits the ability to accurately reconstruct internal climate variability both regionally and globally. By detrending the individual data points in the satellite altimetry prior to creating the basis functions for the reconstruction, we reconstruct only the variability about the long-term, background trend. It should be noted that the background trend that is not represented will contain not only the long-term increase in sea level associated with a warming climate, but also the GMSL contribution of climate variability that is not captured by the reconstruction. The second difference is found in reconstructing the Indo-Pacific and Atlantic regions separately, and then recombining into a global reconstruction. In general, variability in the Indo-Pacific dominates the global basis functions used to perform the reconstruction. This tends to lead to a poor representation of sea level in the Atlantic and surrounding areas. Here, we divide the globe into two separate regions, one containing the Indo-Pacific and the other containing the Atlantic. Two separate reconstructions are performed, with the two datasets subsequently merged to form the global reconstruction used here.

To estimate the dominant decadal trend pattern in the sea level, linear trends over ten-year windows were computed at every location across the globe from the sea level reconstruction spanning from 1900 through 2014. Incrementing by one year at a time, this resulted in 104 ten-year trend maps. An empirical orthogonal function (EOF) decomposition of these trend maps was then performed to determine the dominant ten-year trend pattern in the sea level reconstruction, similar to the analysis done in Hamlington *et al*. [2013], although with a different window size. As a test of the stationarity of the spatial pattern, the dominant EOF was computed over the time-periods from 1900 to 1950 and from 1950 to 2000. The resulting EOF patterns exhibited strong agreement with the EOF discussed in this paper computed over the full time period. To find corresponding patterns in TWS, precipitation and SSS, ten-year trend patterns were similarly computed from each dataset. The EOF PC time series in Fig. 1E was then projected onto the ten-year trend patterns of each dataset to create spatial patterns in the respective variables (Fig. 1B–D).
